# Pain processing and pain assessment in Huntington’s disease: Study protocol of the Huntington’s disease - PAIN study

**DOI:** 10.1371/journal.pone.0346039

**Published:** 2026-04-10

**Authors:** Gregory P. Sprenger, Monique van Velzen, Wilco P. Achterberg, Raymund A.C. Roos, Susanne T. de Bot

**Affiliations:** 1 Department of Neurology, Leiden University Medical Center, Leiden, the Netherlands; 2 Amstelring, Huntington Center, Amsterdam, the Netherlands; 3 Department of Anaesthesiology, Leiden University Medical Center, Leiden, the Netherlands; 4 Department of Anaesthesiology, Erasmus Medical Center, Rotterdam, the Netherlands; 5 Department of Public Health and Primary Care, Leiden University Medical Center, Leiden, the Netherlands; 6 Topaz Huntington Center Overduin, Katwijk, The Netherlands; University of Pecs Medical School, HUNGARY

## Abstract

**Background:**

Huntington’s disease (HD) is an autosomal dominant neurodegenerative disease that causes motor (*e.g.,* chorea) and non-motor symptoms (*e.g.,* neurocognitive, neuropsychiatric and autonomic disturbances). Pain is a frequently reported non-motor symptom in HD, with a prevalence of around 40% in the manifest stage. Despite its high prevalence, patients with HD seem to be at risk for undertreatment of pain, as reflected by the lower analgesic use in HD in the advanced stages. The relatively low use of analgesics may result from under-recognition and inadequate assessment of pain, particularly in the advanced stages of HD. As HD progresses, pain recognition and assessment become increasingly challenging due to the emergence and progression of motor and neurocognitive symptoms. In contrast, the high prevalence of pain may be attributable to disturbances in the pain processing in terms of endogenous pain inhibition or pain facilitation. Despite the availability of specifically developed and internationally standardized experimental pain assessments to test psychometrics properties of observational pain scales and disturbances in pain processing, studies using these pain assessments in HD are too limited to permit definitive conclusions.

**Methods:**

A cross-sectional experimental study will be conducted in twenty genetically and clinically confirmed adult-onset HD patients. The primary aim is to assess the feasibility of the experimental design, which comprises three different standardized pain assessments, using a predefined feasibility checklist.

**Discussion:**

If feasibility is demonstrated, future studies using the comprehensive experimental design, including individually tailored experimental painful stimuli, are expected to provide insight into the psychometric properties of the Pain Assessment in Impaired Cognition scale (PAIC15) and the potential demonstration of altered pain processing in HD, supporting the development of improved pain management regimens. In particular, the motor symptoms (*e.g.,* facial chorea) of HD may have an adverse impact on the psychometric properties of the PAIC15.

**Trial registration:**

Trial registration on ClinicalTrials.gov with number: NCT06693466. Medical Research Ethics Committee Leiden, The Hague and Delft registration number: P24.014.

## 1. Introduction

Huntington’s disease (HD) is an autosomal dominant neurodegenerative disease, caused by an increased number of cytosine-adenine-guanine (CAG) repeats on the short arm of chromosome 4 in the DNA sequence*,* the gene that encodes huntingtin (*HTT*) [1]. The *HTT* gene codes for the protein huntingtin, which is essential for normal neural development, however, its precise function remain incompletely understood [2].The resulting abnormally elongated polyglutamine repeat in the Huntingtin protein causes neuronal loss in the brain, particularly in the striatum [3,4]. The estimated incidence is 0.48 cases per 100,000 person-years, and the prevalence is approximately 5 per 100,000 [5]. HD is characterized by progressive involuntary movements (*e.g.,* chorea), neurocognitive impairments and neuropsychiatric changes, with a reduced life expectancy of 15–20 years from diagnosis. Besides this well-known triad of symptoms and signs in HD, also other non-motor symptoms in HD are described such as weight loss, sleep disturbances, autonomic changes and systemic symptoms [6,7]. Pain is another important non-motor symptom in HD, with a prevalence of around 40% in the manifest HD stage [8–11]. Patients with HD seem to be at risk for undertreatment of pain in the advanced stages [9,10].

Pain management regimens rely on the use of valid and reliable pain measurements. To date, the use of self-reported pain scales is the gold standard in pain assessment [12,13]. These self-reported pain scales may, however, be less reliable in the advanced stages of HD, because of the potential presence of (major) neurocognitive disturbances [12,13]. In the advanced stages, communication disabilities and neurocognitive disturbances are likely to be profound, potentially interfering with self-reporting of pain. Therefore, observational pain scales, which rely on observing pain behaviour (*e.g.,* facial expression, body movements and vocalization), might bypass this issue [12,13]. A potential feasible observational pain scale is the Pain Assessment in Impaired Cognition (PAIC15) [14,15]. The PAIC15 is a multidisciplinary, internationally developed, tested, and consensus-based meta-tool for pain assessment, comprising the most valid and reliable items drawn from the best previously established observational pain scales [14–18]. Recent studies demonstrated promising results concerning the interrater reliability of the PAIC15 between observers from different countries (Denmark, Germany, Italy, Israel and Spain) and neurocognitive disorders (mild cognitive impairment, dementia, HD, and intellectual disabilities) [14,19]. In these particular studies, however, the intensity of the (non-) painful stimuli (delivered by a hand-held pressure algometer) was not individually tailored, and the severity of the (non-) motor symptoms of HD was not further specified [14,19]. Subsequently, potential individual differences in the intensity of pain and the severity of (non-) motor symptoms might be present, that can significantly affect the pain experience and hence the facial expression on pain stimuli. Furthermore, given the small sample size of patients with HD in these studies [14,19], drawing definitive conclusions regarding the reliability of the PAIC15 in HD would be premature. Concerning its validity in HD, to our knowledge, no studies have yet been conducted. To assess the psychometric properties (*e.g.,* validity and reliability) of the PAIC15 in HD, a standardized experimental pain assessment with a different type of stimulus (e.g., heat), individually tailored and assessed in well-defined HD groups, is needed.

Fundamental knowledge of HD's effect on pain processing and perception is absolutely needed to improve the pain management regimens. To our knowledge, the available studies addressing the effect of HD on pain processing are scarce, include small sample sizes and have methodological shortcomings. An experimental study using Laser Evoked Potentials (LEPs) demonstrated significantly slower and weaker nociceptive processing in HD compared to healthy controls [20,21]. Another small experimental study, measuring the nociceptive withdrawal reflex (NWR) of the biceps femoris, which is a measure for endogenous pain inhibition, showed no differences in pain inhibition in patients with HD compared to healthy controls [22]. In the aforementioned studies, however, the HD groups were not clearly defined for the different stages of the disease. Furthermore, the intensity of the stimulus to elicit the NWR, was not individually tailored by using the subjects’ subjectively experienced pain. This may cause different pain experiences in terms of intensity between subjects, causing potentially biased results.

Internationally standardized experimental pain assessments are available to assess the psychometric properties of an observational pain scale, such as the PAIC15, and to gain fundamental knowledge about the effect of HD on pain processing [23–25]. The primary aim of the current study is to assess the feasibility of three different internationally standardized experimental pain assessments in HD, in one single comprehensive study.

For this study, twenty genetically and clinically confirmed adult-onset HD (AoHD) patients will be included. From medical ethical perspectives, methodological point of view and the lack of comparable studies in HD assessing the feasibility of these specific standardized experimental pain assessments, only patients in the early and middle stage of HD will be included in this pilot study.

## 2. Materials and methods

### 2.1. Objectives

The main objective of this study is to assess the feasibility of three commonly used and internationally standardized experimental pain assessments in patients with HD. If the experimental pain assessments are deemed feasible to conduct in HD, the study will be expanded to include more patients and healthy controls in order to assess the exploratory objectives as primary objectives. An amendment request will then be submitted to the local Medical Research Ethics Committee (MREC).

In current study the following exploratory objectives are included: 1) To assess the psychometric properties (test-retest, inter- and intra-rater reliability and measurement error) of the facial expression, body movements and vocalization items of the Pain Assessment in Impaired Cognition scale (PAIC15), in patients with HD. 2) To assess the effect of HD on pain processing in terms of the facial response to pain, and 3) To assess the prevalence and extent of pain inhibition and facilitation (representing the endogenous pain modulation system) in early and middle stage HD. 4) To make an initial estimate of the measurement error (*i.e.,* agreement), stated as the systematic and random error of a patients’ score that is not attributed to true changes in the construct to be measured [26,27], for each endpoint related in current study to pain processing in HD (*e.g.,* the endogenous pain modulation outcomes).

### 2.2. Design

In order to achieve our study objectives, in total twenty genetically and clinically confirmed adult-onset HD (AoHD) patients will be included. Participants will be recruited at the Huntington outpatient Center of the department of Neurology at the Leiden University Medical Center (LUMC), and by an advertisement in the magazine of the Dutch Patients Association for HD (in Dutch: ‘Vereniging van Huntington’). The participants will be informed about the study rationale and procedures, and a written consent is required to participate in the study.

**Fig 1:**
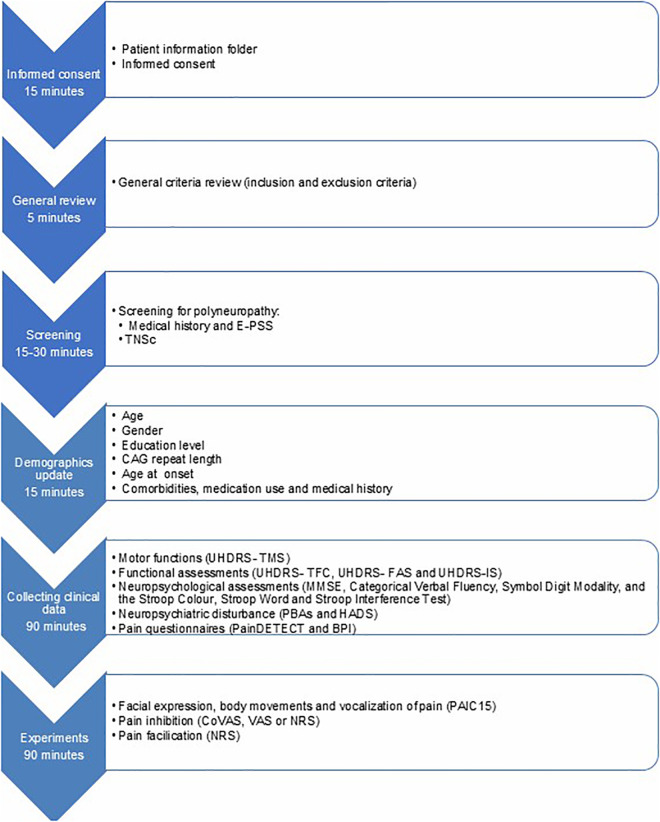
Flowchart of the HD-PAIN study. Brief Pain Inventory (PBI), Computerized Visual Analogue Scale (CoVAS), Erasmus Polyneuropathy Symptom Score (E-PSS), Function Assessment Scale (FAS), Hospital Anxiety and Depression Scale (HADS), Independence Scale (IS), Mini-Mental State Examination (MMSE), Numerical Rating Scale (NRS), Pain Assessment in Impaired Cognition scale (PAIC15), Problem Behaviours Assessment – short (PBAs), Total Functional Capacity (TFC), Total Motor Score (TMS), Total Neuropathy Score-clinical (TNSc), Unified Huntington Disease Rating Scale (UHDRS), Visual Analogue Scale (VAS).

After reviewing the inclusion and exclusion criteria by the research team, the investigator assesses the presence of somatosensory dysfunction and, in particular, symptoms of a sensorimotor neuropathy. In the absence of interfering disturbances in the somatosensory systems, the clinical and experimental data will be collected (Fig 1). Otherwise, the participant will not proceed with further study assessments. The collection of the clinical and experimental data can be done on one day or planned on two different days, depending on what is most convenient for the participant. The clinical data are validated clinical HD tests, e.g., the Unified HD Rating Scale- Total Functional Capacity or Total Motor Score (UHDRS-TFC or TMS), frequently used in HD research [28]. The TFC score of the UHDRS will be used to define the HD groups. A UHDRS-TFC score of 11–13 (stage 1) corresponds to HD group 1, whereas a TFC score of 3–10 (stages 2 and 3) corresponds to HD group 2 (see also paragraph 2.5.2) [28–30].

In short, the experimental setup consists of three blocks, whereby in the first block, data will be collected of the facial expression, body movements and vocalization of non-painful and painful heat stimuli. After that, data for testing the endogenous pain inhibition using conditioned pain modulation and pain facilitation (*i.e.,* temporal summation) will be collected in the second and third block, respectively (see for more details, paragraph 2.6).

The stimuli will be induced using the Medoc Thermal Sensory Analzer® (TSA2) with a 3x3 cm thermode probe (first and second block), a cold water immersion (using Lauda waterbath, only second block) and a set of seven pinprick stimulators (only third block). The devices used for inducing the stimuli have safety limits, so no physical harm can be inflicted on the patients.

The entire study is approved by the accredited Medical Research Ethics Committee Leiden (MREC), The Hague and Delft (the Netherlands) that gave its approval on 7 March 2024 (registration number: P24.014). A written informed consent will be obtained. In addition, the study is registered in the trial registration on ClinicalTrials.gov with number: NCT06693466.

### 2.3. Power calculations

The primary aim of this pilot study is to assess the feasibility of three different internationally standardized experimental pain assessments in stages 1, 2 and 3 of HD, therefore a power calculation was not considered necessary. A convenient sample of twenty patients will be included, with ten patients allocated to the HD1 group (*e.g.,* stage 1) and ten patients to the HD2 group (stage 2 and 3). The sample size is based on similar feasibility studies [31,32].

### 2.4. Participants

Inclusion criteria: 1) Genetically and clinically confirmed adult-onset HD patients (AoHD) (clinical onset ≥ 21 years, CAG repeats ≥ 36; Diagnostic Confidence Level [DCL] of 4]); in stage 1, stage 2 and 3, according to the Shoulson-Fahn system [29,30], 2) Good general health apart from having HD, 3) Able to give written informed consent.

Exclusion criteria: 1) Juvenile and Paediatric HD (age at onset <21 years), 2) Patients in the late stage of the disease (UDHRS-Total Functional Capacity [TFC] score < 3), 3) Having medical, psychiatric, or other conditions (other than HD) that, according to the investigator, may compromise the patient’s ability to understand the patient information sheet, to give informed consent, to comply with all study requirements, or to perform study assessments, 4) Having a history of (in the past year) or current (ab)use of any drug, alcohol or medication that, in the opinion of the investigator, may seriously interfere with the primary objectives of the study, 5) The presence of a sensorimotor neuropathy (Total Neuropathy Score – clinical ≥ 7) or any other disturbance significantly disturbing the somatosensory systems, based on medical history and/or clinical examination, that can interfere with the experimental pain assessments, 6) Women who are pregnant or breastfeeding.

All criteria related to HD and its severity are based on established international standards [33]. The exclusion criterion for sensorimotor neuropathy was determined based on internal consensus between the Departments of Neurology and Anesthesiology.

In addition, five health care professionals experienced with HD working in specialized HD nursing homes, will be included in this study to score the PAIC15 based on video material (see for more details, paragraph 2.6). PAIC15 assessors must complete the freely available e-learning of the PAIC15 and be able to present the certificate before taking part in the study (see link: www.paic15.com). The PAIC15 is translated into Dutch and validated [34].

### 2.5. Measurement outcomes

#### 2.5.1. Demographic data.

The following demographic data will be collected: age, sex, educational level (according to International Standard Classification of Education [ISCED]), CAG-repeat length, comorbidities, and concomitant medication use (dose and indication), according to International Statistical Classification of Disease and Related Health Problems classification (ICD-10) and Anatomical Therapeutic Chemical (ATC) codes, respectively [35,36].

#### 2.5.2. Clinical data.

The motor symptoms of HD are represented by the score of the Total Motor Score of the Unified Huntington’s Disease Rating Scale (UHDRS-TMS). Scores range from 0 (no symptoms) to 124 (severe symptoms) [28]. The functional assessments include the UHDRS Total Functional Capacity (TFC) scale, Function Assessment Scale and the Independence Scale to assess the general functioning [28]. Total Function Capacity (TFC) of the Unified Huntington Disease Rating Scale (UHDRS) will be used to define the HD groups: UHDRS-TFC score 11–13 (stage 1), 7–10 (stage 2), 3–6 (stage 3), 1–2 (stage 4) and a score of 0 (stage 5) [29,30].

Exploratory analyses will also present the data according to the Huntington’s Disease – Integrated Staging System (HD-ISS) [33]. Due to the absence of essential imaging data required to determine the stage of patients in HD, a promising algorithm capable to bypass this issue will be used [37].

The neuropsychological assessments will include the Mini-Mental State Examination (MMSE) [38], Categorical Verbal Fluency, Symbol Digit Modality Test (SDMT), and the Stroop Colour, Stroop Word and Stroop Interference Test. These tests are all part of the UHDRS rating system, commonly used in HD, with a focus on processing speed, attention and executive function [28]. The MMSE is a paper-based cognitive screening test with a maximum score of 30. A score under the cut point of 24 indicates the presence of neurocognitive impairments [39]. The Categorical Verbal Fluency examines the ability to spontaneously produce words within a certain category (*e.g.,* animals) orally within a fixed time (60 seconds). The SDMT involves a substitution task, whereby the subject has 90 seconds to pair specific numbers with given geometric figures. The Stroop Colour and Word Test involves naming colours (*e.g.,* red, green, blue) and reading the words for colours in black ink. The Stroop Interference test involves reading words of colours (*e.g.,* red, green, blue) where the word colour is written in a different colour ink.

The Problem Behaviours Assessment – short (PBAs) and the Hospital Anxiety and Depression Scale (HADS) will be scored for assessing the neuropsychiatric disturbances. The PBAs is semi-structured interview and measures frequency and intensity of neuropsychiatric symptoms, typically seen in HD [40]. It compromises of eleven symptoms of HD: depression, suicidal ideation, anxiety, irritability, aggressive behaviour, apathy, perseveration, obsessions and compulsions, paranoid thinking, hallucinations and disorientation. The HADS is a self-report rating scale and offers a brief rating of depression and anxiety symptoms [41]. It includes 14 items and each item is rated on a four-point scale. The cut off score is ≥ 8 for anxiety and depression [42].

The presence of pain will be assessed by using the painDETECT and the pain interference items of the Brief Pain Inventory (BPI) [43–45]. The painDETECT is a patient-based screening questionnaire for neuropathic pain components and has been validated in Dutch. It includes questions about the perceived intensity of pain, the grading of pain, the pain course and whether or not the pain is radiating. A score above 19, indicates the presence of neuropathic pain.

The BPI is a short self-report questionnaire, validated in Dutch, which allows participants to rate the severity of their pain both at the current day and over the last 24 hours, as well as the degree to which their pain has interfered with their feelings and functioning over the last 24 hours. For this study, only the pain interference items of the BPI will be used.

### 2.6. Experimental setup

#### 2.6.1. First block: Facial expression of pain.

**Fig 2:**
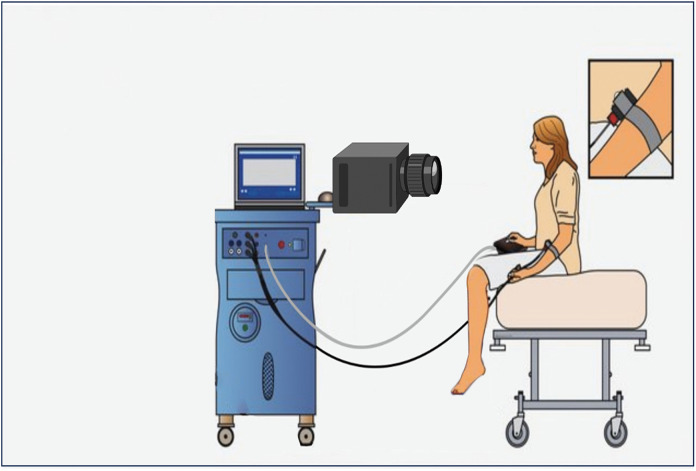
An illustration of the experimental setup for block 1. A heat thermode will apply the thermal stimuli on the volar side of the lower arm. The video camera will record the facial expression, the body movements and vocalization of pain. Reprinted from (Sprenger, G.P. 2025. Prevalence and burden of pain in Huntington’s disease) under a CC BY license, with permission from (Sprenger, G.P.: Leiden University Medical Center), original copyright (2025).

In the **first block** a commonly used standardized experimental pain assessment will be used to assess the facial expression, body movements and vocalization of pain (Fig 2) [23]. The first block includes a pre-experimental and an experimental phase.

#### 2.6.2. Pre- experimental phase.

In the pre-experimental phase, the heat pain threshold will be determined by using the method of limits. The baseline temperature of the thermode will be set at 35°C and will be increased until the participant feels a first pain sensation (Numerical Rating Scale [NRS] = 1 [stated as barely painful]). In line with other studies, the cooling and heating rate is set at 0.7°C/s, but can be decreased (to 0.5°C/s) to account for a delayed reaction time [46]. After familiarization, there are four trials, and the average of the temperature of the last three trials will be used to determine the pain threshold.

Secondly, the intensity (i.e., temperature) of a moderate painful stimulus will be determined for each participant. The cooling and heating rate is also set at 0.7°C/s and can be decreased to 0.5°C/s. The target is a temperature producing a score of pain of 7 out of 10 on the NRS. It is determined by the average of three representative tests. The score of 7 out of 10 on the NRS was selected to induce facial response in the majority of the subjects, without causing sensitization and reaching the subjects’ tolerance [47].

#### 2.6.3. Experimental phase.

**Fig 3:**
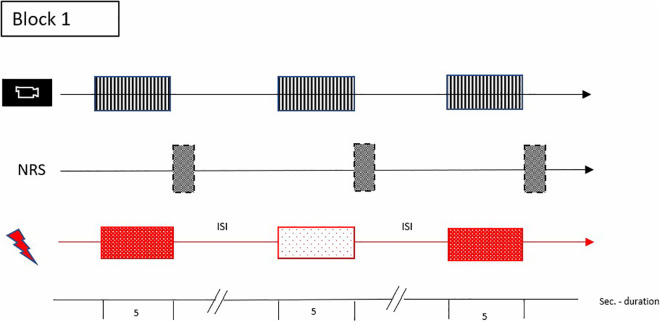
The experimental step-ups for block 1. Inter- Stimulus- Interval (ISI), non-painful stimuli 
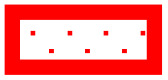
. Numerical Rating Scale (NRS) 
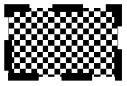
, painful stimuli 
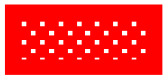
, Recoding facial expression/ body movements/ vocalization 
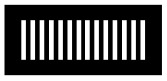
, Seconds (Sec.) – duration.

In the experimental phase, participants will be pseudo- at randomly exposed to eight pre-set non-painful (3°C below pain threshold) and eight moderately painful (NRS of 7) stimuli (Fig 3). The non-painful condition will serve as a reference for ‘baseline’ facial expression, body movements and vocalization. In practice, the temperature will increase from baseline temperature (35°C) to either the pre-set individual non-painful stimulus or the pre-set moderate painful stimulus (with NRS of 7), subsequently remaining at a plateau for 5 seconds. The rate of change between painful and non-painful stimuli is 5°C/s and to prevent sensitization the inter-stimulus-interval (ISI) is 25 seconds.

#### 2.6.4. General procedure.

The individually tailored stimuli will be applied in a pseudo- at random, blinded order to each participant. During the experimental stage, facial expressions, body movements and vocalizations in response to the stimuli will be recorded using a video camera, allowing for retrospective scoring of these items (Fig 2). To diminish biases, two independent raters will score the facial expression items of the PAIC15. In case the two reviewers failed to reach consensus in the scores, a third reviewer will be consulted.

The NRS will be scored by the subject after each stimulus, to double check whether or not the stimuli (non-painful and painful) is still sufficient in terms of intensity [23]. A mean post-test NRS score of ≥ 6 and ≤ 8 will be accepted as a painful stimulus for proceeding the tests. The (non-) painful stimuli will be applied to the volar side of the non-dominant lower arm, which will be divided into three zones. The thermode will be moved from one zone to the other zone between the stimuli. As already mentioned, some settings can be adjusted during the experiment if necessary (Table 1).

**Table 1:** Summary of the standard technical settings of the pre- and experimental stage and the potential adjustments of the settings in block 1.

	Standard setting	Potential adjustment
*Pre-experimental phase*		
Cooling and heating rate	0.7 °C/s	To 0.5 °C/s
*Experimental phase*		
Cooling and heating rate	5°C/s	To 2.5°C/s
Inter-stimulus-interval	25 seconds	Maximum of 60 seconds
Amount of stimuli	8 non-painful and 8 painful stimuli	4 non-painful and 4 painful stimuli

#### 2.6.5. Second block: Conditioned pain modulation.

**Fig 4:**
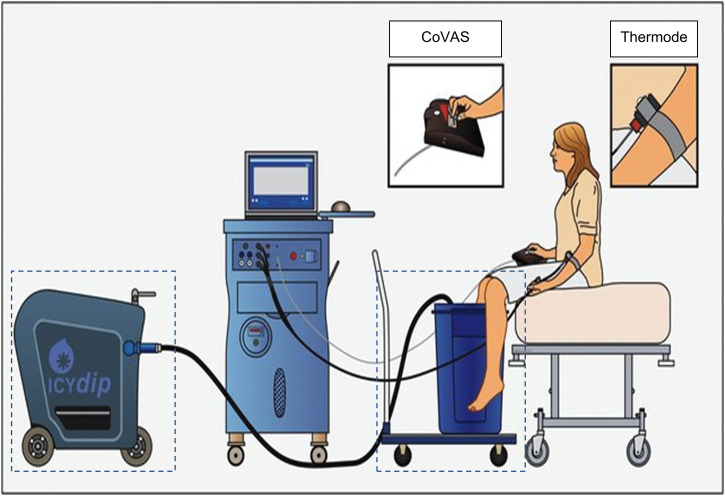
An illustration of the experimental setup for block 2. A heat thermode will apply the thermal stimuli on the volar side of the lower arm. The dotted lines represent the water bath cooling system, which will deliver the conditioning stimulus to the subject during **block 2**. Computerized Visual Analogue Scale (CoVAS). Reprinted from (Niesters, M. 2014. Evolution of endogenous analgesia) under a CC BY license, with permission from (Niesters, M.: Leiden University Medical Center), original copyright [2014].

The standardized experimental pain assessment that will be used in the **second block**, to assess endogenous condition pain modulation (CPM) (*i.e.,* pain inhibition system), is according to internationally agreed standards [24,25]. The CPM will be assessed by using a heat stimulus as the test stimulus and a cold water bath immersion as a conditioning stimulus (Fig 4). Like for Block 1, the setup includes a pre-experimental and an experimental phase.

#### 2.6.6. Pre-experimental phase.

Before assessing the CPM, the temperature of the test stimulus will be determined for each participant. The baseline temperature of the thermode will be set at 35°C and the heating rate is set at 1.5 C/s and the cooling rate at 6°C/s). The temperature of the test stimulus is defined as the temperature producing a peak pain level of 60 out of 100 mm on the Computerized Visual Analogue Scale (Co)VAS and is determined by three representative tests of 10 seconds each (including familiarization). For the conditioning stimulus, the temperature of the water will be set at 4°C. The intensity of the test and conditioning stimuli are according to international standards [25].

#### 2.6.7. Experimental phase.

**Fig 5:**
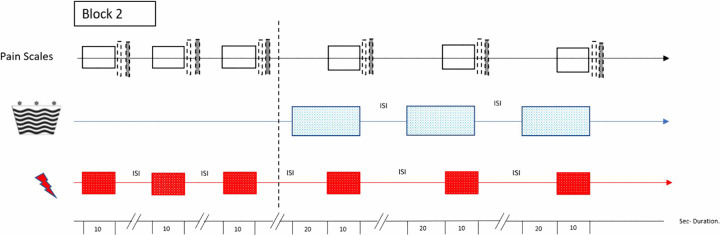
The experimental step-up for block 2. Cold water (conditioning stimulus) 
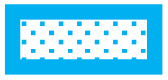
, Computerized Visual Analogue Scale (CoVAS) 
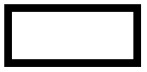
, Inter- Stimulus- Interval (ISI). Numerical Rating Scale (NRS) 
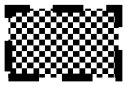
, Painful test stimulus 
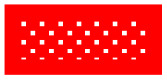
, Visual Analogue Scale (VAS) 
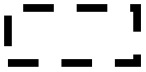
, Seconds – Duration (Sec.).

The experimental phase consists of two parts, including of three tests each. The first part, includes only the test stimulus and in the second part the test and conditioning stimulus will be given simultaneously (Fig 5).

For assessing CPM, the temperature of the thermode is increased from 35°C (baseline temperature) to the individualized pre-set temperature, with a plateau duration of 10 seconds, after which the temperature returns to baseline. The heating rate is set at 1.5°C/s and cooling rate at 6°C/s. The conditioning stimulus will be applied 20 seconds prior to the start of the test stimulus and will simultaneously end with the test stimulus. An interval of 5 minutes will be taken between the each test. During the experiment, subjects continuously report the pain intensity level of the test stimulus by using a slider on a computerized potentiometer that ranges from 0 (no pain) to 100 mm (worst pain imaginable), which allows continuous, electronic monitoring of the VAS (CoVAS). For assessing the conditioned pain modulation, the CoVAS and VAS will be used. In case the CoVAS or VAS are not feasible to use, the NRS will be used to determine the temperature of the test stimulus and to test the CPM [25]. If written pain reporting is also too difficult for the subject, a verbal rating may be given that will be transcribed by the researcher to a written NRS. The relative difference in pain perception in conditioned versus unconditioned stimuli is a measure of CPM.

#### 2.6.8. General procedure.

The test stimulus will be applied to the volar side of the non-dominant lower arm, while the conditioning stimulus consists of an immersion of the contralateral foot in cold water. To prevent sensitization, a 5 minute rest period will be taken between tests and the volar side of the forearm will be divided into three zones. The thermode will be moved from one zone to the other zone between the stimuli. Pain perception of the conditioning stimulus will be collected by verbal rating using the NRS. Potential setting changes during the experiment also apply to this block (Table 2).

**Table 2:** Summary of the standard technical settings of the pre- and experimental designs and the potential adjustments of the settings in block 2. Computerized Visual Analogue Scale (CoVAS), Numerical Rating Scale (NRS), Visual Analogue Scale (VAS).

	Standard setting	Potential adjustment
*Pre-experimental phase*		
Baseline temperature – conditioning stimulus	4 °C	To 6 °C
Heating rate test stimulus	1.5 °C/s	To 1.0 °C/s
*Experimental phase*		
Heating rate test stimulus	1.5 °C /s	To 1.0 °C/s
Rest period	5 minutes	To 10 minutes
Exposure to test and conditioning stimuli	Total of 6 blocks, 3 including the conditioning stimuli	Total of 4 blocks, 2 including the conditioning stimuli
Self-reported pain scale	CoVAS and VAS	Written NRS or verbal NRS

#### 2.6.9. Third block: Pain facilitation.

In the **third block,** pain facilitation will be assessed, and therefore the magnitude of the pain induced by a mechanical pinprick stimulus is compared to that of a train of 10 pinprick stimuli of the same force repeated at a 1/s rate (weight of pinprick: weight that is mildly painful during mechanical pain sensitivity test (NRS 1–2). The participant will be asked to verbally rate the pain on a scale of 0–10 (where 0 is no pain and 10 the worst pain imaginable) after the single stimulus and again after the train of stimuli. In total, three rounds will be executed. A set of seven pinprick stimulators can be used (8, 16, 32, 64, 128, 256 and 512 mN). The test stimulus will be applied at the volar side of the forearm.

### 2.7. Experimental endpoints

The facial expression, body movements and vocalization domain items of the PAIC15 will be scored. All three domains comprise of five items with a five-point Likert scale (Not at all, Slight degree, Moderate degree, Great Degree, Not scorable) [14,15].

Pain modulation is measured by CoVAS and VAS scores or, if not feasible, by the NRS. The relative difference in pain perception in conditioned versus unconditioned stimuli is a measure of CPM. The pain facilitation will be measured using the NRS, which will also be used to set the pain threshold.

### 2.8. Statistical analysis

#### 2.8.1. Primary study parameter.

To determine whether the different experimental pain assessments are feasible in patients with HD, a screening tool was developed consisting of dichotomized items (S1 Appendix). Each item of the screening tool represents a key component of one of the experimental pain assessment. For each item, a predefined cut-off score ranging from 60% to 80% was determined, indicating the minimum score required to deem that specific component of the assessment feasible. The included items are, among others, comprehension of the instructions, minimum percentage of patients who can complete the test(s) and specific settings of the pain assessments (S1 Appendix). For each participant, feasibility will be assessed separately for each component of the pain assessment. Based on the aggregated item scores, an average score will be calculated for each pain assessment separately to also assess the overall feasibility of the respective pain assessment.

In addition, a modified post-test questionnaire will be administered to each participant and health care professional to assess the feasibility of the pain assessment, specifically regard to the participants’ burden of the pain assessments and the health care professionals’ assessment of the quality of the video material for PAIC15 scoring(S1 Appendix) [48]. Both questionnaires consists of dichotomized items (response options: ‘yes’ or ‘no’) and a minimum score of ≥ 60% was predefined for each item to consider the respective component feasible. For each item in the questionnaires, the final score will be calculated as the proportion of affirmative responses across all participants who completed the questionnaire.

#### 2.8.2. Exploratory study parameters.

The psychometric properties (test-retest, inter- and intra-rater reliability and measurement error) of the facial expression, body movements and vocalization items of the PAIC15 will be assessed. Different statical analyses for the reliability and measurement error (*i.e.,* ‘agreement’) can be considered, depending on the type of scale (*e.g.,* continuous or categorical) (Table 3) [49]. Regarding the statistical analyses for a categorical scale, it is considered more appropriate to use a percentage agreement instead of Cohen’s kappa to assess the probability of another rater giving the same answers [50].

**Table 3:** Overview of the study outcome, type of scale and analysis [49].

Outcome	Scale		Type of scale	Analysis	
				Reliability	Measurement error
Psychometric properties	PAIC15	Facial expression, body movements and vocalization items	Categorical	ICC or (weighted) Kappa	% agreement
		Total Score PAIC15	Continuous	ICC	ICC, SEM and Limits of agreement

Intraclass correlation coefficients (ICC), Pain Assessment in Impaired Cognition scale (PAIC15), Standard Error of Measurement (SEM).

Secondly, the facial response on pain will be assessed by using the facial expression items of the PAIC15 [14,15]. The facial expression items of the PAIC15 will be scored in terms of frequency (yes/no) (absolute frequency, relative frequency and the percentages of activity during the painful segments), the intensity (four point scale) and an overall score. As demonstrated by other studies [23,51], the data of the facial expression items of the PAIC15 may not be normally distributed. Group differences in terms of the frequency scores of the facial expression items will be calculated using χ^2^ test. The group differences in the overall facial expression of pain, will be analysed using non-parametric or parametric test when applicable.

Thirdly, for the endogenous pain modulation systems, pain inhibition and facilitation, measured sensory responses will be described (VAS, VAS over time, area-under-the-VAS-curve and NRS). Group differences for CPM and pain facilitation will be analysed using non-parametric or parametric tests when applicable. An impaired CPM is defined as less than 10–15% pain inhibition and abnormal pain facilitation is defined as at least two points difference on the NRS, between a single prick and 10 [24,52,53].

Finally, an initial estimate of the measurement error (*i.e.,* agreement) will be tested for each endpoint related in current study to the endogenous pain modulation system [26,27]. The type of analyses depends on the type of scale (*e.g.,* continuous or categorical) (Table 4).

**Table 4:** Overview of the study outcome, type of scale and analysis for assess the measurement error (agreement) [49].

Outcome	Scale	Type of scale	Analysis – measurement error
Pain inhibition	CoVAS	Continuous	SEM and Limits of agreement
	VAS	Continuous	SEM and Limits of agreement
	NRS	Continuous	SEM and Limits of agreement
Pain facilitation	NRS	Continuous	SEM and Limits of agreement

Area-under-the-Curve (AUC); Computerized Visual Analogue Scale (CoVAS), Numerical Rating Scale (NRS), Pain Assessment in Impaired Cognition scale (PAIC15), Standard Error of Measurement (SEM), Visual Analogue Scale (VAS).

In future studies that designate exploratory objectives as primary objectives and formally assess group differences, analyses should be adjusted for relevant sociodemographic and clinical variables. The current study will provide insight into which variables are important to adjust for. The data collected in the pilot study can be fully utilized if a subsequent study is conducted.

### 2.9. Dissemination plan

Results of the feasibility study will be published in a peer-reviewed journal. In addition, the results will be demonstrated during HD congresses, like the European Huntington Disease Network Plenary meetings.

### 2.10. Timeline

Participant recruitment has started in the last quarter of 2025 and will be completed by May 2026. The study timeline is presented in Tables 5 and 6.

**Table 5:** Timeline of the study.

Year	2025				2026			
Quarter	1	2	3	4	1	2	3	4
Data collection			x	x	x	x		
Data analysis						x		
Publication of data							x	x

**Table 6:** Timeline of the study.

	STUDY PERIOD		
	Enrollment	Allocation	Post-allocation
**Timepoint**	-t_1_	0	t_1_
Enrollment:			
Informed consent	x		
Eligibility screen	x		
Screening	x		
Demographic update	x		
Allocation		x	
Assessments:			
Clinical data			x
Experimental data			x

### 2.11. Data management plan

The data management plan outlined by Feleus et al. [54] has been adopted in the present study. Consequently, data will be recorded on paper forms and combined in an electronic database, Castor Electronic Data Capture. Medical information that is important for this study will be retrieved from and/ or cross-checked in the electronic patient file. If a subject does not have a patient file at the including site, details on their medical history can be retrieved from the treating medical specialist or general practitioner, after given consent. All data is encrypted and pseudonymised with a unique participant identification code. The de-pseudonymization code is only available to a limited number of authorized investigating personnel. Names of personnel who have access to the password-protected, coding file, will be listed in a separate file. All the data, also the videos of the facial expression, body movements and vocalization to the stimulus, will be stored for 15 years in accordance with the Dutch Act on Implementation of the General Data Protection Regulation (in Dutch: ‘Uitvoeringswet Algemene Verordening Gegevensbescherming’ (UAVG)), the European Union General Data Protection Regulation (GDPR) and the privacy statements regulations of the LUMC.

Monitoring will take place according to the monitoring plan in collaboration with the internal monitoring department of the LUMC. Scientific rules and regulations will be taken into account. In order to perform quality assessments and in line with the current legislation, de-anonymized data is accessible to the internal LUMC monitor and to regulatory national bodies such as the Dutch Health and Youth Care Inspectorate (in Dutch: ‘Inspectie Gezondheidszorg en Jeugd’).

## 3. Discussion

Pain is an important symptom in HD, with a mean prevalence of around 40% [8–10]. Risk for undertreatment with analgesics may be present, in particular in the advanced HD stages [9,10]. For adequate pain management regimes, the use of reliable (observational) pain scales and fundamental knowledge of HD's effect on pain processing are essential.

To our knowledge, the current study is the first of its kind to assess, in one single comprehensive study design, the feasibility of commonly used and internationally standardized experimental pain assessments in HD. If feasibility is demonstrated, the comprehensive experimental design provides a unique opportunity to assess both the psychometric properties of the PAIC15 and enhance fundamental knowledge of HD on pain processing. Subsequently, this could potentially advance the clinical field by improving the recognition and assessment of pain in patients with HD, ultimately supporting better pain management regimens. Given the motor, neurocognitive and neuropsychiatric symptoms in HD, practical and operational challenges may arise in current study. The HD related symptoms might interfere with completion of the study (too demanding), comprehension of instructions, the use of the different self-reported pain scales and the specific settings of the experiment setups. Among other potential hurdles, the subjects’ experience of the study will be objectively assessed by the developed screening tool (S1 Appendix). Overall, we expect these issues will be of limited impact, since these experimental pain assessments are according to the international agreed standards and also frequently used in different patient populations [55–58]. Furthermore, ad hoc adjustments can be made, providing the possibility to develop the most efficient, qualitative, HD-tailored and least burdensome experimental setup.

It’s good to realise that there may be other unexpected matters beyond those we anticipate, which could be crucial for the feasibility of the different experiment setups. If such matters arise, they will be addressed and published so future studies can anticipate them. Furthermore, extrapolating the findings in current study regarding the feasibility and the results related to the psychometric properties of the PAIC15 to patients in the advanced stages, should be drawn with caution. Conducting these experiments are, however, methodologically and ethically inappropriate in individuals in the advanced stages of HD due to severe cognitive, communicative, and motor impairments to obtain valid informed consent and reliable response patterns. Experimental studies conducted in early and middle stages of HD are therefore essential to assess the psychometric properties of an experimental pain scale such as the PAIC15 and for improving our understanding of whether HD affects pain processing. The feasibility of the pain assessments must, however, be assessed first. In particular, the motor symptoms (*e.g.,* facial chorea and dystonia) of HD may have an adverse impact on the psychometric properties of the PAIC15. Due to the limited number of participants included in the current study, an accredited MREC approved extension of the study in the future is necessary to eventually assess the psychometric properties of the PAIC15 and the effect of HD on pain processing. Finally, future studies assessing the psychometric aspects, particularly the inter- and intra-rater reliability, of the PAIC15 in an advanced care setting for patients with HD are also warranted [26].

## Supporting information

S1 Appendix Screening tool. (PDF)
