# Supplementary material for: Pain processing and pain assessment in Huntington’s disease: Study protocol of the Huntington’s disease - PAIN study
Source: PLoS One. 2026 Apr 10;21(4):e0346039. doi: 10.1371/journal.pone.0346039 (PMC13068213; doi:10.1371/journal.pone.0346039)
Supplement: S1 Appendix — (PDF) [file pone.0346039.s001.pdf]

Supporting Information - Appendix I. Screening tool to assess the feasibility

Item	Endpoint	Cut-offs	
**Qualitative assessment**		HD 1<br>(N = 10)	HD 2<br>(N = 10)
Recruitment of participant		≥ 30%, one year	
Screen failure		≤50%	
Completion of the study		≥ 60%	≥ 50%
Comprehension of the instructions – overall		≥ 70%	≥ 60%
Block 1.	*Pre-experimental phase* (for more details, see Table 1)		
	Cooling and heating rate (0.7 °C/s and 0.5°C/s)	≥ 70%	≥ 60%
	Experimental phase (for more details, see Table 1)		
	Cooling and heating rate (5°C/s or 2.5°C/s)	≥ 70%	≥ 60%
	Inter-stimulus-interval	≥ 70%	≥ 60%
	Amount of stimuli (8 or 4 blocks)	≥ 70%	≥ 60%
	General aspects		
	Subject is able to look straight forward	≥ 70%	≥ 60%
	Tolerate heat pain stimulus (70/100 [NRS])	≥ 70%	≥ 60%
	Comprehension of the Numerical Pain Rating Scale (NRS)	≥ 70%	≥ 60%
	Post-test NRS score of ≥ 4 and ≤ 8	≥ 60%	≥ 70%
	Overall	≥ 70%	≥ 60%
Block 2.	*Pre-experimental phase* (for more details, see Table 2)		
	Baseline temperature – conditioning stimulus	≥ 70%	≥ 60%

	Cooling and heating rate	≥ 70%	≥ 60%
	Experimental phase (for more details, see Table 2)<br>Cooling and heating rate<br>Rest period<br>Exposure to test stimuli – amount (6 or 4 blocks)	 ≥ 70%<br>≥ 70%<br>≥ 70%	 ≥ 60%<br>≥ 60%<br>≥ 60%
	General aspects<br>Tolerate heat pain stimulus (40-60/100 [NRS])<br>Tolerate conditioned stimulus - 4°C or 6°C<br>Comprehension Computerized- Visual Analogue Scale (CoVAS)<br>Comprehension Visual Analogue Scale (VAS)<br>Comprehension Numerical Rating Scale (NRS)	 ≥ 70%<br>≥ 70%<br>≥ 70%<br>≥ 70%<br>≥ 70%	 ≥ 60%<br>≥ 60%<br>≥ 70%<br>≥ 70%<br>≥ 70%
	Overall	≥ 70%	≥ 65%
Block 3.	Tolerate mechanical pain stimulus	≥ 80%	≥ 80%
Post-test questionnaire after each **block** - Subjects	Do you think the experiment was feasible?		
	a) Doable ? Y/N	≥ 70%	≥ 50%
	b) Easy to complete? Y/N	≥ 70%	≥ 50%
	c) Easy to comprehend the instructions? Y/N	≥ 80%	≥ 80%
	d) Easy to execute the instructions? Y/N	≥ 80%	≥ 80%
	e) Did you experience enough time to report your pain experience?	≥ 60%	≥ 60%
	f) Did you experience any difficulties in using the electronical device to report your pain experience? Y/N	≥ 70%	≥ 60%

	g) Do you believe the test is safe and not harmful to people? Y/N	≥ 90%	≥ 90%
	h) Any suggestions to improve the experiment?		
Total time elapsed for the experiment			
Block 1.		≥ 50% within 45 minutes	
Block 2.		≥ 50% within 45 minutes (wb)	
Block 3.		≥ 50% within 10 minutes	
Ceiling or floor effect concerning the heat pain threshold and/ or test/ conditioning stimulus		≤ 20%	≤ 20%
Post-test questionnaire – <u>Health care professionals</u>	a) The quality of the videos is good? Y/N	≥ 60%	≥ 60%
	b) The face is clearly visible? Y/N	≥ 60%	≥ 60%
	c) The lighting of the videos is good? Y/N	≥ 60%	≥ 60%
	d) The time for scoring the items of the PAIC15 is sufficient? Y/N	≥ 60%	≥ 60%
	e) There are no other distracting stimuli on the videos? Y/N	≥ 60%	≥ 60%

Supporting information - Appendix I. Huntington's Disease (HD); No (N); Yes (Y); Pain assessment in Impaired Cognition scale (PAIC15); without break (wb)
